# Spermidine protects against ETEC-induced intestinal injury by reshaping the gut microbiota and activating the AhR/IL-22 axis

**DOI:** 10.3389/fmicb.2026.1907051

**Published:** 2026-08-25

**Authors:** Peng Jiang, Zhao Ling, Shuo Han, Jing Wang

**Affiliations:** 1 College of Animal Science and Technology, Tarim University, Alar, China; 2 State Key Laboratory for Diagnosis and Treatment of Severe Zoonotic Infectious Diseases, College of Veterinary Medicine, Institute of Zoonosis, Jilin University, Changchun, China; 3 Engineering Laboratory of Tarim Animal Diseases Diagnosis and Control, Xinjiang Production & Construction Corps, Alar, China; 4 Hangzhou Xiaoshan Donghai Aquaculture Co. Ltd., Hangzhou, Zhejiang, China

**Keywords:** aryl hydrocarbon receptor, enterotoxigenic *Escherichia coli*, gut microbiota, IL-22, intestinal barrier, spermidine

## Abstract

**Background:**

Enterotoxigenic *Escherichia coli* (ETEC) is a major cause of infectious diarrhea, characterized by excessive inflammatory responses and intestinal barrier disruption. Spermidine (SPD), a naturally occurring polyamine, has been implicated in intestinal homeostasis; however, its protective effects and underlying mechanisms against ETEC-induced intestinal injury remain unclear.

**Methods:**

A mouse model of ETEC K88-induced intestinal injury was established to evaluate the protective effects of SPD. Disease severity, histopathological alterations, inflammatory responses, intestinal permeability, and epithelial barrier integrity were assessed. The protective mechanism of SPD was further investigated using IPEC-J2 cells, pharmacological inhibition of aryl hydrocarbon receptor (AhR), 16S rRNA gene sequencing, and fecal microbiota transplantation (FMT).

**Results:**

SPD administration significantly alleviated ETEC-induced intestinal injury in mice, as evidenced by reduced body weight loss, diarrhea severity, histopathological damage, systemic inflammation, and intestinal permeability, together with restoration of tight junction integrity. In ETEC-challenged IPEC-J2 cells, SPD improved cell viability, suppressed pro-inflammatory cytokine production, and restored the expression of AhR, CYP1A1, ZO-1, and occludin. Mechanistically, inhibition of AhR signaling by CH-223191 markedly weakened the protective effects of SPD and reduced CYP1A1 and IL-22 expression. Furthermore, 16S rRNA sequencing revealed that SPD reshaped the gut microbiota, while FMT from SPD-treated donors transferred protective effects to recipient mice. AhR inhibition further attenuated the benefits mediated by microbiota transplantation.

**Conclusion:**

These findings demonstrate that SPD protects against ETEC-induced intestinal injury through coordinated regulation of gut microbiota remodeling and activation of the AhR/CYP1A1/IL-22 signaling axis. This study provides mechanistic insights into the role of SPD in maintaining intestinal barrier homeostasis and highlights its potential as a nutritional intervention strategy against enteric bacterial infections.

## Introduction

Enterotoxigenic *Escherichia coli* (ETEC) is a major enteric pathogen responsible for diarrheal diseases in humans and animals worldwide. In humans, ETEC represents one of the leading bacterial causes of acute watery diarrhea, particularly among children under 5 years of age living in low-resource settings and international travelers. Previous epidemiological studies have estimated that ETEC contributes substantially to the global burden of childhood diarrheal diseases, resulting in considerable morbidity and mortality (von Mentzer and Svennerholm, 2024; Zhang et al., 2022). Beyond its impact on human health, ETEC is also an important pathogen in livestock production, causing neonatal diarrhea in calves and post-weaning diarrhea in piglets, thereby leading to significant economic losses in the agricultural sector (Sun and Kim, 2017). Following intestinal colonization, ETEC expresses fimbrial adhesins and secretes enterotoxins, including heat-labile toxin (LT) and heat-stable toxin (ST), which disrupt epithelial barrier integrity, increase intestinal permeability, and trigger excessive inflammatory responses, ultimately resulting in intestinal dysfunction and diarrhea (Fu et al., 2024; Xia et al., 2015). Therefore, understanding the mechanisms underlying ETEC-induced intestinal injury and identifying safe interventions to enhance host intestinal defense remain important priorities.

Spermidine (SPD) is a naturally occurring polyamine ubiquitously present in living organisms and dietary sources (Madeo et al., 2020). Increasing evidence has demonstrated that SPD participates in diverse biological processes, including cellular metabolism, immune regulation, oxidative stress resistance, and maintenance of intestinal homeostasis (Eisenberg et al., 2009; Madeo et al., 2018). Recent studies have suggested that SPD exerts beneficial effects in experimental models of intestinal inflammation by preserving epithelial barrier function and modulating host immune responses (Niechcial et al., 2023). However, whether SPD protects against ETEC-induced intestinal injury and the molecular mechanisms underlying its action remain largely unknown.

The aryl hydrocarbon receptor (AhR) is a ligand-activated transcription factor that plays a pivotal role in coordinating mucosal immunity and epithelial barrier maintenance. Activation of AhR promotes downstream signaling events, including induction of CYP1A1 and production of IL-22, thereby enhancing epithelial integrity and tissue repair (Gronke et al., 2019; Monteleone et al., 2011). Notably, accumulating evidence indicates that gut microbiota-derived metabolites can serve as endogenous AhR ligands, linking microbial ecology with host immune regulation (Miyamoto et al., 2024). These observations raise the possibility that modulation of the gut microbiota may represent an upstream mechanism through which SPD influences AhR signaling and intestinal health. Nevertheless, direct evidence connecting SPD-induced microbiota remodeling with AhR activation during ETEC infection is currently lacking.

In the present study, we investigated the protective effects of SPD against ETEC-induced intestinal injury using both murine and intestinal epithelial cell models. Furthermore, by integrating gut microbiota profiling, pharmacological inhibition of AhR, and FMT, we sought to elucidate the mechanistic interplay among SPD, the gut microbiota, and AhR signaling. Our findings demonstrate that SPD alleviates ETEC-induced intestinal inflammation and barrier dysfunction by remodeling the gut microbiota and activating the AhR/CYP1A1/IL-22 pathway, providing new mechanistic insights into the use of dietary polyamines as potential interventions for enteric infectious diseases.

## Materials and methods

### Chemicals and reagents

Spermidine (SPD, HY-B1776) was purchased from MedChemExpress (Monmouth Junction, NJ, USA). CH-223191 (CH, C10093) was obtained from Psaitong (Beijing, China). Commercial ELISA kits for the quantification of IL-1β, IL-6, TNF-α, IFN-γ, IL-22, D-lactate, and diamine oxidase (DAO) in mouse serum, as well as IL-8 in cell culture supernatants, were purchased from Jonlnbio Industrial Co., Ltd. (Shanghai, China). The ELISA kit used for TNF-α determination in cell culture supernatants was obtained from Neobioscience Technology Co., Ltd. (Shenzhen, China).

### Bacterial strain and culture condition

The ETEC K88 strain (serotype O149:K91:K88ac) was obtained from our laboratory stock collection. The strain was routinely cultured in Luria–Bertani (LB) broth at 37 °C with continuous shaking at 220 rpm overnight. Prior to infection, the overnight culture was diluted 1:100 in fresh LB broth and incubated at 37 °C until reaching the logarithmic growth phase. Bacterial cells were harvested by centrifugation at 4,000 × *g* for 10 min, washed twice with sterile phosphate-buffered saline (PBS), and resuspended in sterile PBS to the desired concentration. The bacterial concentration was determined by measuring optical density and confirmed by plate counting on LB agar plates. Fresh bacterial suspensions were prepared immediately before each inoculation and adjusted to the required concentration for animal infection experiments.

### ETEC-induced intestinal injury model and spermidine administration

**Figure 1:**
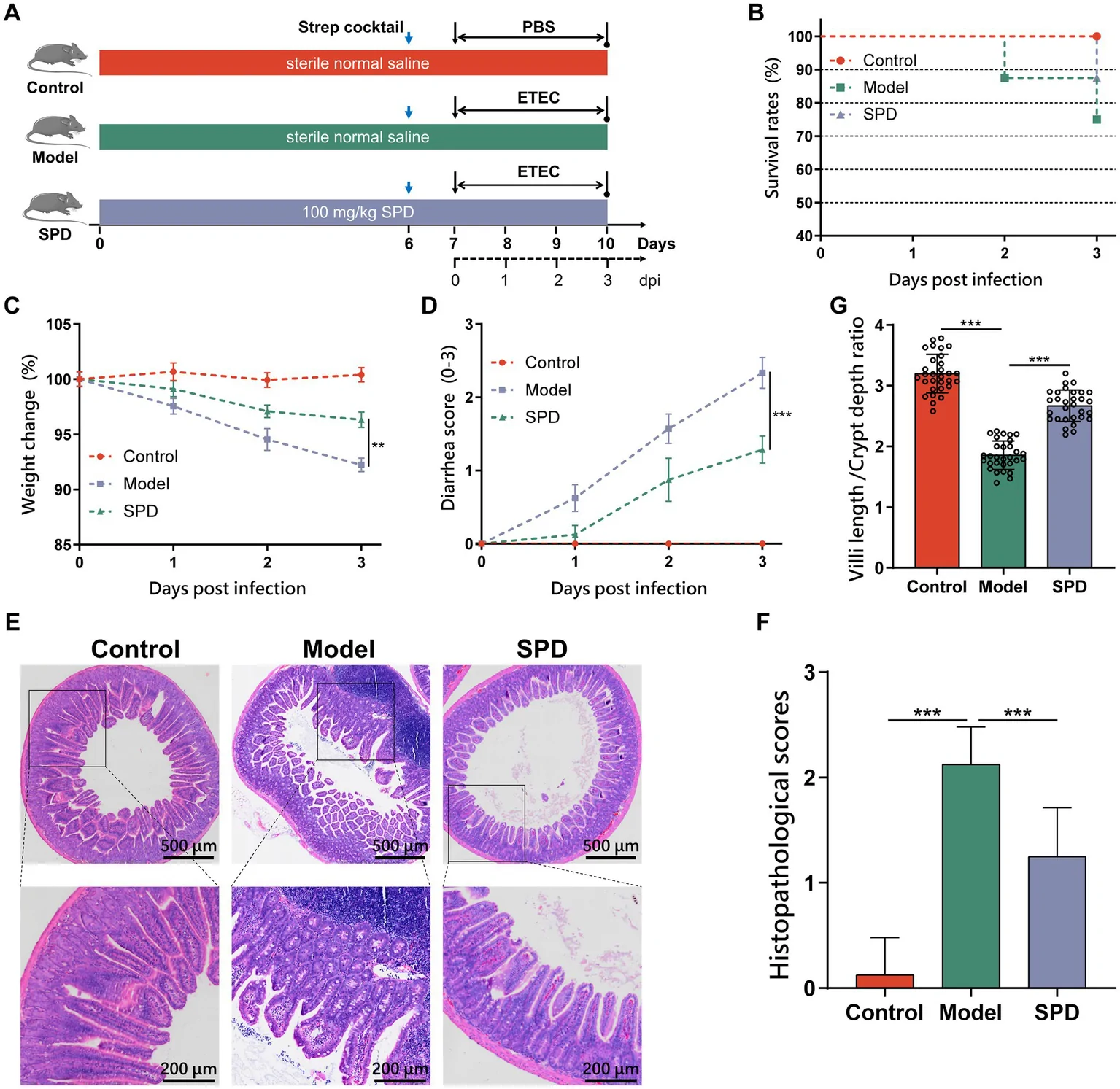
Spermidine alleviates ETEC-induced intestinal injury and improves clinical outcomes in mice. **(A)** Schematic illustration of the experimental design and spermidine (SPD) administration protocol in the murine ETEC infection model. **(B)** Kaplan–Meier survival curves of mice following ETEC challenge. **(C)** Changes in body weight during the infection period. **(D)** Diarrhea scores recorded after ETEC infection. **(E)** Representative hematoxylin and eosin (H&E)-stained sections of the jejunum from each group. Upper panels show low-magnification views (scale bar = 500 μm), and lower panels show corresponding higher-magnification images of the boxed regions (scale bar = 200 μm). **(F)** Histopathological injury scores of jejunal tissues. **(G)** Quantification of the villus height-to-crypt depth (V/C) ratio. Data are presented as mean ± SD unless otherwise indicated. Statistical significance was determined by one-way or two-way ANOVA followed by Tukey’s multiple-comparison test, and survival curves were analyzed using the log-rank (Mantel–Cox) test. **p < 0.01, ***p < 0.001.

Pathogen-free female C57BL/6 mice (6–8 weeks old) were obtained from Changsheng Biotechnology (Liaoning, China). All mice were kept under specific pathogen-free conditions and individually housed in the same temperature- and humidity-controlled room on a 12-h light/dark cycle with ad libitum access to feed and water throughout the experimental period. In experimental for ETEC infection and SPD intervention (Experimental 1, Figure 1A), mice were randomly assigned to three groups (*n* = 8): Control, ETEC model (Model), and spermidine treatment (SPD) groups. To facilitate ETEC colonization, mice were administered streptomycin sulfate (5 g/L) in drinking water 1 day prior to bacterial challenge to disrupt the resident intestinal microbiota. For spermidine treatment, mice in the SPD group were orally administered spermidine (100 mg/kg BW) for 7 consecutive days before ETEC infection and continued to receive the same dose throughout the infection period. Mice in the Control and Model groups received an equal volume of sterile saline. ETEC infection was performed according to previously described methods with minor modifications (Lyu et al., 2025). Briefly, mice in the Model and SPD groups were orally challenged with 200 μL freshly prepared ETEC K88 suspension (1 × 10^9^ CFU/mL) once daily for three consecutive days. Mice in the Control group received an equal volume of sterile PBS.

To investigate whether the protective effects of spermidine against ETEC-induced intestinal injury are mediated through activation of the AhR pathway, a second animal experiment (Experimental 2) was performed. Mice were randomly assigned to four groups (*n* = 8): Model, Model + CH-223191 (CH + Model), SPD, and SPD + CH-223191 (CH + SPD). The ETEC infection procedure and spermidine administration regimen were identical to those described in Experimental 1. Briefly, mice in the SPD and CH + SPD groups received spermidine (100 mg/kg BW) by oral gavage throughout the experimental period. To inhibit AhR signaling, mice in the CH + Model and CH + SPD groups were intraperitoneally injected with the AhR antagonist CH-223191 (10 mg/kg BW) once daily during the entire experimental period (Cao et al., 2024), including the 7-day pretreatment phase and the subsequent 3-day ETEC infection period. Mice in the Model and SPD groups received an equivalent volume of vehicle (5% DMSO + 40% PEG300 + 5% Tween-80 + 50% saline).

To investigate whether the protective effects of spermidine were transferable through the gut microbiota and whether AhR signaling was involved in this process, fecal microbiota transplantation (FMT) (Experimental 3) was conducted. Recipient mice were randomly assigned to three groups (*n* = 8): FMT-Model, FMT-SPD, and CH + FMT-SPD. Before transplantation, recipient mice received a broad-spectrum antibiotic cocktail in drinking water for 7 consecutive days to deplete the resident gut microbiota (Yin et al., 2026). Fresh fecal samples were collected from donor mice in the Model and SPD groups described in Experimental 1. Briefly, fecal pellets were pooled within each group, suspended in sterile PBS (100 mg feces/mL), thoroughly homogenized, and freshly prepared for transplantation. Mice in the FMT-Model and FMT-SPD groups received the corresponding donor microbiota once daily throughout the experimental period, including the 7-day pretreatment phase and the subsequent 3-day ETEC infection period. To inhibit AhR signaling, mice in the CH + FMT-SPD group received the same FMT regimen as the FMT-SPD group and were intraperitoneally injected with CH-223191 once daily during the entire experimental period. Following microbiota colonization, all recipient mice were challenged with ETEC K88 as described above.

Body weight, clinical symptoms, and disease activity were monitored throughout the experimental period. At 3 days post ETEC challenge, mice were deeply anesthetized with 1% sodium pentobarbital (50 mg/kg body weight, intraperitoneal injection) and subsequently euthanized by cervical dislocation. Blood, intestinal tissues, and fecal samples were then collected for subsequent analyses.

### Analysis of clinical outcomes

Clinical outcomes, including body weight and diarrhea severity, were evaluated at 0, 1, 2, and 3 days post-infection (dpi). Body weight was recorded daily and expressed as the percentage change relative to the initial body weight. Diarrhea severity was assessed using a previously described scoring system with minor modifications (Ledwaba et al., 2020). Briefly, fecal consistency was scored as follows: 0, well-formed pellets; 1, soft stools adhering to the wall of the collection tube; 2, pasty stools with or without mucus; 3, watery stools with or without mucus; and 4, stools containing visible blood. Diarrhea incidence and severity were monitored throughout the experimental period.

### Histopathological evaluation

Jejunal tissues were fixed in 4% paraformaldehyde for 24 h at room temperature, dehydrated through a graded ethanol series, cleared in xylene, and embedded in paraffin. Paraffin-embedded tissues were sectioned at 4 μm thickness and stained with hematoxylin and eosin (H&E) according to standard procedures. Morphometric analysis was performed by measuring villus height and crypt depth from thirty well-oriented villus–crypt units per sample using ImageJ software (National Institutes of Health, Bethesda, MD, USA). The villus height-to-crypt depth ratio (V/C ratio) was subsequently calculated. Histopathological alterations were evaluated in a blinded manner using a semi-quantitative scoring system as previously described with minor modifications (Wong et al., 2015). Histological damage was graded on a scale of 0–3 according to the severity of epithelial injury and inflammatory changes: score 0, normal intestinal architecture without pathological alterations; score 1, mild lesions characterized by villus shortening, partial loss of crypt architecture, mild inflammatory cell infiltration, epithelial vacuolization, and submucosal edema; score 2, moderate lesions characterized by marked villus blunting, crypt degeneration or focal necrosis, moderate inflammatory cell infiltration, epithelial vacuolization, and edema; score 3, severe lesions characterized by extensive villus destruction, crypt necrosis, pronounced inflammatory cell infiltration, severe edema, vacuolization, and involvement of the muscular layer accompanied by neutrophil infiltration. Higher scores indicated more severe intestinal tissue damage.

### Evaluation of intestinal permeability

Intestinal permeability was evaluated using a 4 kDa fluorescein isothiocyanate-dextran (FD4, Sigma-Aldrich, St. Louis, MO) assay (Zhang et al., 2024). Briefly, six mice per group were orally administered 200 μL FD4 solution (40 mg/mL; Sigma–Aldrich, St. Louis, MO, USA) 4 h before sacrifice. Serum FD4 fluorescence was measured using a Cytation 5 Cell Imaging Multi-Mode Reader (BioTek, Winooski, VT, USA) at excitation and emission wavelengths of 485 and 535 nm, respectively. Elevated serum FD4 levels were considered indicative of increased intestinal permeability.

### Enzyme-linked immunosorbent assay (ELISA)

The concentrations of inflammatory cytokines in serum samples and cell culture supernatants, as well as serum levels of D-lactate and diamine oxidase (DAO), were determined using commercially available ELISA kits according to the manufacturers’ instructions. Briefly, appropriately diluted samples and standards were added to 96-well microplates pre-coated with capture antibodies and incubated under the recommended conditions. After sequential washing steps, substrate solution was added for color development, and the reaction was terminated with stop solution. Absorbance was measured at 450 nm using a Synergy LX multimode microplate reader (BioTek). The concentrations of the analytes were calculated from standard curves generated using the corresponding kit standards.

### Western blot analysis

Total proteins from intestinal tissues and cultured cells were extracted using RIPA lysis buffer (Thermo Fisher Scientific, Carlsbad, CA, USA) supplemented with protease inhibitor cocktail. Protein concentrations were determined using a BCA Protein Assay Kit (Solarbio, Beijing, China). Equal amounts of protein (30 μg per sample) were separated by SDS-PAGE and subsequently transferred onto polyvinylidene fluoride (PVDF) membranes (Millipore, Billerica, MA, USA). The membranes were blocked with 5% bovine serum albumin (BSA; YEASEN, Shanghai, China) in TBST for 2 h at room temperature and then incubated overnight at 4 °C with the indicated primary antibodies. The primary antibodies used for Western blot were TLR4 (Proteintech, Cat No. 66350-1-Ig, 1:4,000), MyD88 (ABclonal, Cat No. A0980, 1:2,000), p-p65 (ABclonal, Cat No. AP1294, 1:2,000), p65 (ABclonal, Cat No. A19653, 1:5,000), GAPDH (Proteintech, Cat No. 60004-1-Ig, 1:20,000), ZO-1 (Proteintech, Cat No. 21773-1-AP, 1:5,000), occludin (Proteintech, Cat No. 66378-1-Ig, 1:5,000), AhR (Proteintech, Cat No. 67785-1-Ig, 1:2,000), and CYP1A1 (Proteintech, Cat No. 13241-1-AP, 1:1,000). HRP-conjugated goat anti-rabbit IgG (Proteintech, Cat No. SA00001-2, 1:5,000) and HRP-conjugated goat anti-mouse IgG (Proteintech, Cat No. SA00001-1, 1:5,000) were used as secondary antibodies. Following three washes with TBST, the membranes were incubated with horseradish peroxidase (HRP)-conjugated secondary antibodies for 1 h at room temperature. Protein bands were visualized using an enhanced chemiluminescence (ECL) detection system and quantified using ImageJ software. Band intensities were normalized to GAPDH and expressed relative to the control group.

### Cell culture and treatment

IPEC-J2 cells were cultured in DMEM/F12 supplemented with 10% FBS and 100 U/mL penicillin, and 100 μg/mL streptomycin at 37 °C in a humidified incubator. To quantify the cytotoxicity of SPD, IPEC-J2 cells were seeded into 96-well plates at a density of 1 × 10^4^ cells per well and treated with increasing concentrations of SPD (0–800 μM). Cell viability was determined using a Cell Counting Kit-8 (CCK-8; GlpBio, USA) according to the manufacturer’s instructions. Based on the cytotoxicity results, concentrations of 25, 50, and 100 μM SPD were selected for subsequent experiments.

The ETEC-induced cellular injury model was established as previously described with minor modifications (Liu and Guo, 2024). Briefly, IPEC-J2 cells were seeded into six-well plates at a density of 5 × 10^5^ cells per well. Upon reaching approximately 70–80% confluence, cells were serum-starved in FBS-free medium for 6 h and subsequently pretreated with SPD (25, 50, or 100 μM) in medium containing 2% FBS for 48 h. Cells were then challenged with ETEC K88 at a multiplicity of infection (MOI) of 100 in antibiotic-free medium containing 2% FBS for 2 h. Following treatment, cells were collected for subsequent analyses.

### Gut microbiota profiling by 16S rRNA gene sequencing

Fresh fecal samples were aseptically collected from mice and immediately stored at −80 °C prior to analysis. Total microbial genomic DNA was isolated using a commercial DNA extraction kit according to the manufacturer’s instructions. The V3–V4 regions of the bacterial 16S rRNA gene were amplified using the universal primer pair 338F/806R. Amplicon libraries were prepared and sequenced on an Illumina high-throughput sequencing platform by Paisennuo Biotechnology Co., Ltd. (Shanghai, China). Raw sequencing data were subjected to quality control, denoising, and sequence assembly using the QIIME2 platform. Amplicon sequence variants (ASVs) were generated using the DADA2 algorithm after removal of low-quality and chimeric sequences. Taxonomic assignment was performed against the SILVA reference database. Microbial community diversity was assessed by alpha-diversity indices and beta-diversity analyses, including principal coordinate analysis (PCoA). Differentially abundant bacterial taxa among groups were identified using linear discriminant analysis effect size (LEfSe), and microbial composition was further compared at different taxonomic levels.

### Statistical analysis

All statistical analyses were performed using GraphPad Prism 8.0 (GraphPad Software, San Diego, CA, USA). Data are presented as the mean ± standard deviation (SD), unless otherwise indicated. For animal experiments, mice were initially allocated to each group (*n* = 8 per group). Due to infection-related mortality in the Model group and occasional attrition in other groups, the final number of animals included in statistical analyses ranged from 5 to 8 per group. Only animals that survived until the predefined endpoint were included in the final analysis. No data were excluded unless animals met predefined humane endpoint criteria or died during the experimental period. *In vitro* experiments were performed with at least three independent biological replicates. Differences among multiple groups were analyzed using one-way analysis of variance (ANOVA) followed by Tukey’s multiple comparisons test. Body weight changes over time were analyzed using two-way ANOVA followed by Bonferroni’s multiple comparisons test. Non-parametric data, including diarrhea scores and histopathological scores, were analyzed using the Kruskal–Wallis test followed by Dunn’s multiple comparisons test. Statistical significance was defined as *p* < 0.05.

## Results

### Spermidine pretreatment protects mice against ETEC-induced intestinal injury and improves clinical outcomes

To investigate whether SPD pretreatment protects against ETEC-induced intestinal injury, a murine infection model was established as illustrated in Figure 1B. During the 3-day post-infection period, no mortality was observed in the Control group, whereas two mice in the Model group died at 2 and 3 dpi, respectively, and one mouse in the SPD group died at 3 dpi. Although survival was only modestly affected, SPD-treated mice exhibited improved clinical manifestations compared with the untreated infected mice. ETEC infection induced a marked reduction in body weight, with mice in the Model group showing an approximate 8% loss by 3 dpi. In contrast, SPD administration significantly attenuated body weight loss, limiting the reduction to approximately 3% (Figure 1C). Consistently, ETEC challenge resulted in a pronounced increase in diarrhea scores throughout the infection period, whereas SPD pretreatment significantly reduced diarrheal severity (Figure 1D), indicating that SPD effectively alleviated the clinical manifestations associated with ETEC infection. Histopathological examination of jejunal sections further demonstrated the protective effects of SPD (Figure 1E). The Control group displayed intact villus architecture and normal mucosal morphology without evident pathological alterations. In contrast, mice in the Model group exhibited severe intestinal damage characterized by extensive epithelial disruption, villus blunting and necrosis, inflammatory cell infiltration, hemorrhage, and partial crypt dilation. These pathological changes were markedly ameliorated in SPD-treated mice, which retained relatively preserved mucosal architecture with substantially reduced tissue injury. Correspondingly, histological injury scores were significantly decreased and the villus-to-crypt (V/C) ratio was significantly increased in the SPD group compared with the Model group (Figures 1F,G). Collectively, these findings demonstrate that SPD pretreatment protects mice against ETEC-induced intestinal injury by improving clinical manifestations, including diarrhea and body weight loss, while preserving intestinal histological integrity.

### Spermidine attenuates ETEC-induced systemic inflammatory responses

**Figure 2:**
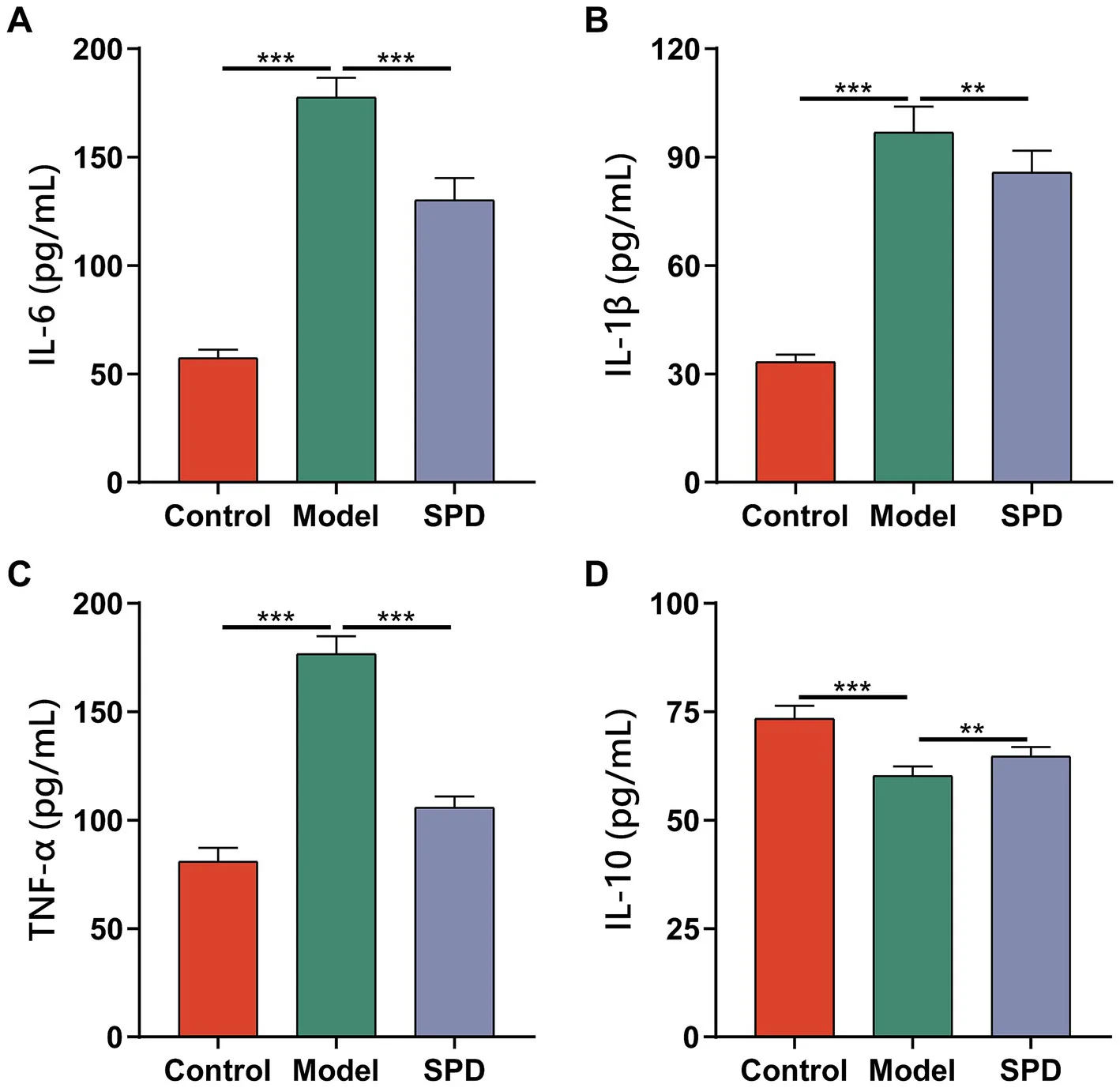
Spermidine attenuates ETEC-induced systemic inflammatory responses in mice. **(A–D)** Serum concentrations of IL-6 **(A)**, IL-1β **(B)**, TNF-α **(C)**, and IL-10 **(D)** were determined by ELISA in Control, Model, and spermidine (SPD)-treated mice following ETEC infection. Data are presented as mean ± SD. Statistical significance was determined by one-way ANOVA followed by Tukey’s multiple-comparison test. **p < 0.01, ***p < 0.001.

To determine whether spermidine modulates the systemic inflammatory response induced by ETEC infection, serum levels of representative pro-inflammatory and anti-inflammatory cytokines were quantified by ELISA. As shown in Figures 2A–D, ETEC challenge triggered a pronounced inflammatory response, as evidenced by significantly elevated serum concentrations of IL-6, IL-1β, and TNF-α compared with the Control group. In contrast, the level of the anti-inflammatory cytokine IL-10 was markedly reduced following ETEC infection. Notably, SPD treatment effectively reversed these alterations by suppressing the production of pro-inflammatory cytokines and restoring IL-10 levels toward those observed in healthy controls. These findings indicate that spermidine attenuates ETEC-induced systemic inflammation and contributes to the re-establishment of immune homeostasis during bacterial infection.

### Spermidine suppresses activation of the TLR4/MyD88/NF-κB signaling pathway in ETEC-infected mice

**Figure 3:**
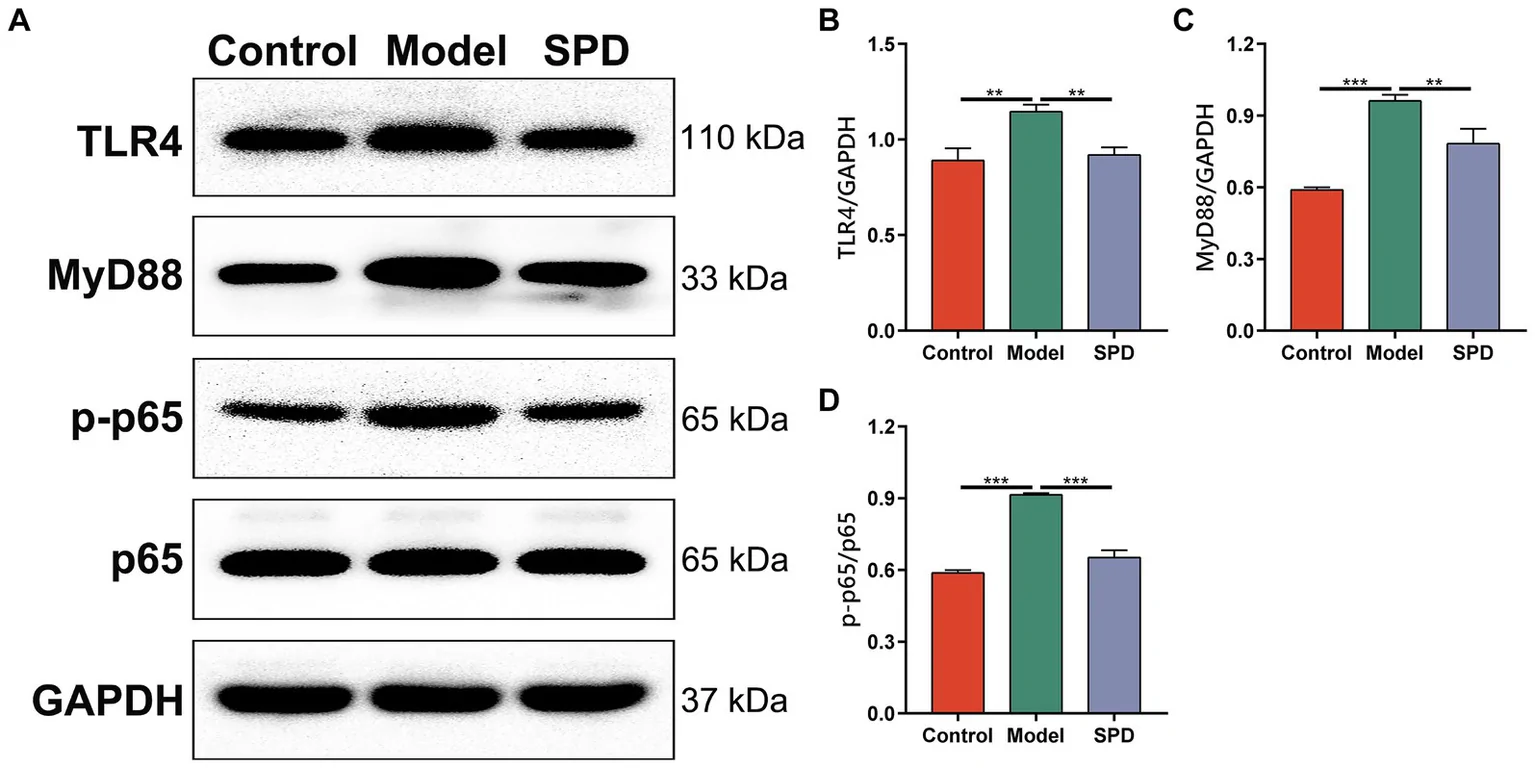
Spermidine suppresses activation of the TLR4/MyD88/NF-κB signaling pathway in ETEC-infected mice. **(A)** Representative Western blot images showing the protein expression of TLR4, MyD88, phosphorylated NF-κB p65 (p-p65), total NF-κB p65 (p65), and GAPDH in jejunal tissues. **(B–D)** Densitometric analyses of TLR4 **(B)**, MyD88 **(C)**, and p-p65 **(D)** protein expression normalized to the corresponding loading controls. Data are presented as mean ± SD. Statistical significance was determined by one-way ANOVA followed by Tukey’s multiple-comparison test. **p < 0.01, ***p < 0.001.

To investigate the molecular mechanisms underlying the anti-inflammatory effects of spermidine, key proteins involved in the TLR4/MyD88/NF-κB signaling pathway were analyzed by Western blotting (Figure 3A). Compared with the Control group, ETEC infection markedly increased the protein expression of TLR4 and MyD88, accompanied by enhanced phosphorylation of NF-κB p65, indicating activation of the canonical TLR4/MyD88/NF-κB signaling cascade. In contrast, spermidine treatment significantly attenuated these changes, as evidenced by reduced expression of TLR4 and MyD88 and a marked decrease in p-p65 levels (Figures 3B–D). These results suggest that spermidine suppresses ETEC-induced inflammatory signaling, at least in part, which is associated with reduced activation of the TLR4/MyD88/NF-κB pathway.

### Spermidine preserves intestinal barrier integrity in ETEC-infected mice

**Figure 4:**
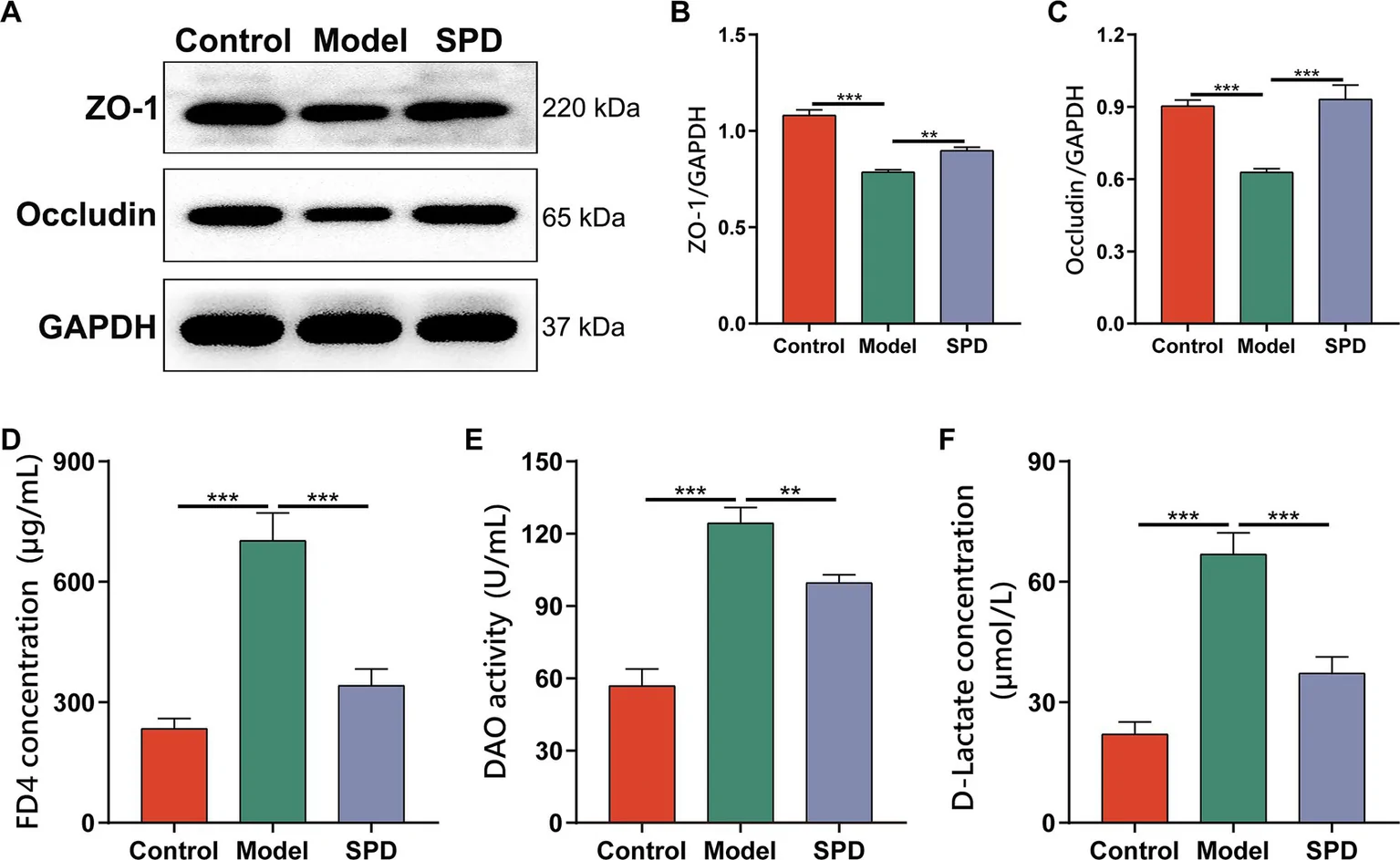
Spermidine preserves intestinal barrier integrity in ETEC-infected mice. **(A)** Representative Western blot images showing the protein expression of ZO-1, occludin, and GAPDH in jejunal tissues. **(B–C)** Densitometric analyses of ZO-1 **(B)** and occludin **(C)** protein expression. **(D–F)** Serum levels of fluorescein isothiocyanate-dextran 4 kDa (FD4) **(D)**, diamine oxidase (DAO) activity **(E)**, and D-lactate **(F)** as indicators of intestinal permeability and barrier function. Data are presented as mean ± SD. Statistical significance was determined by one-way ANOVA followed by Tukey’s multiple-comparison test. **p < 0.01, ***p < 0.001.

To further determine whether spermidine protects intestinal barrier function during ETEC infection, the expression of tight junction proteins and serum biomarkers of intestinal permeability were evaluated. Western blot analysis showed that ETEC challenge markedly reduced the protein expression of ZO-1 and occludin compared with the Control group, indicating disruption of epithelial tight junction integrity. Notably, spermidine treatment significantly restored the expression of both tight junction proteins (Figures 4A–C). Consistent with these findings, ETEC infection significantly increased serum FD4 levels, DAO activity, and D-lactate concentrations, reflecting impaired intestinal barrier function and enhanced intestinal permeability. In contrast, spermidine administration markedly reduced serum FD4, DAO, and D-lactate levels relative to the Model group (Figures 4D–F). Collectively, these results demonstrate that spermidine effectively preserves intestinal barrier integrity and alleviates ETEC-induced intestinal hyperpermeability.

### Spermidine protects IPEC-J2 cells against ETEC-induced injury and restores AhR/CYP1A1 signaling

**Figure 5:**
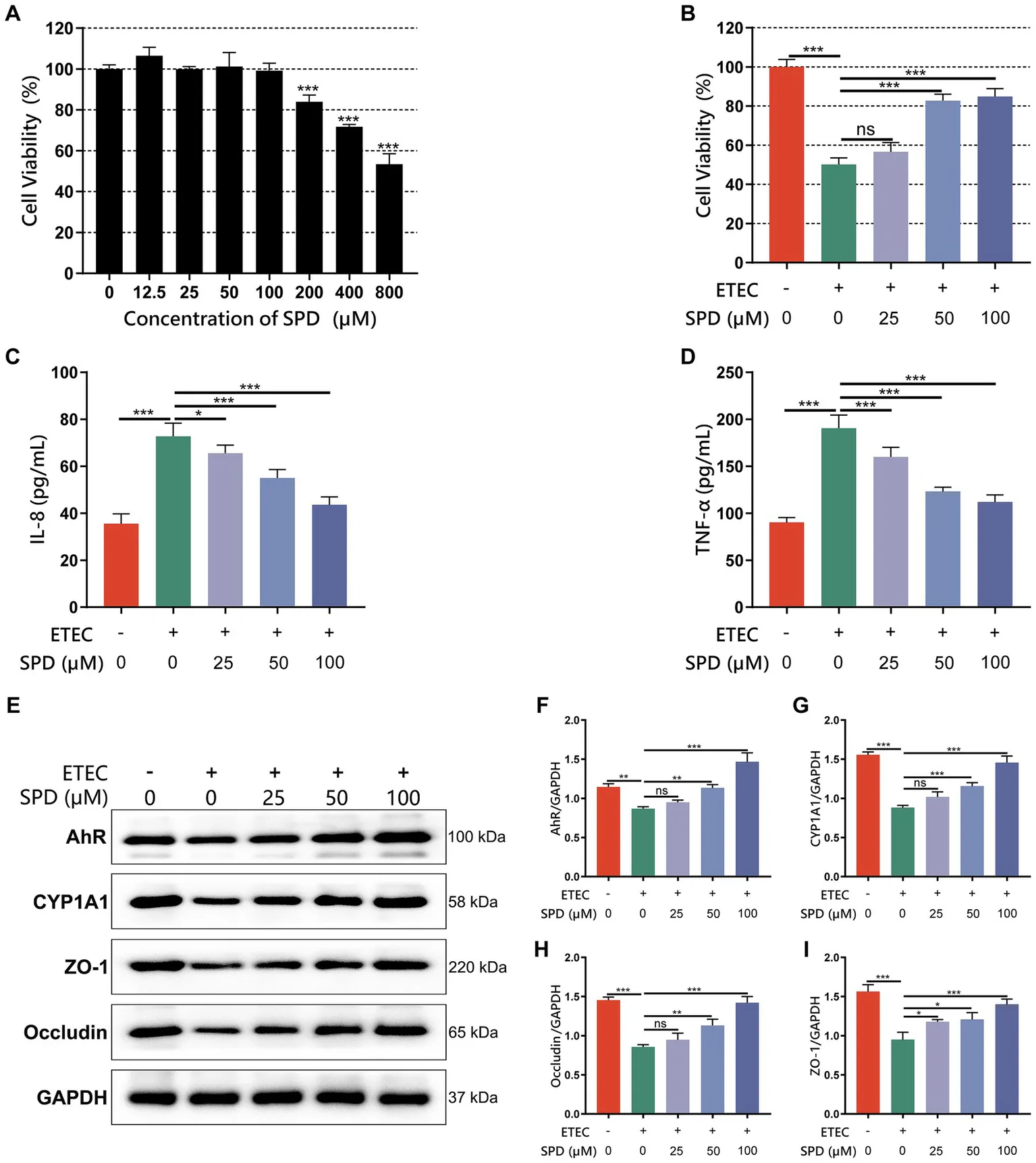
Spermidine protects IPEC-J2 cells against ETEC-induced injury and restores AhR/CYP1A1 signaling. **(A)** Cell viability of IPEC-J2 cells treated with increasing concentrations of SPD (0–800 μM) determined by the CCK-8 assay. **(B)** Cell viability of ETEC-infected IPEC-J2 cells following treatment with different concentrations of SPD (25, 50, and 100 μM). **(C–D)** Concentrations of IL-8 **(C)** and TNF-α **(D)** in culture supernatants measured by ELISA. **(E)** Representative Western blot images showing the protein expression of AhR, CYP1A1, ZO-1, occludin, and GAPDH. **(F–I)** Densitometric analyses of AhR **(F)**, CYP1A1 **(G)**, ZO-1 **(H)**, and occludin **(I)** protein expression. Data are presented as mean ± SD. Statistical significance was determined by one-way ANOVA followed by Tukey’s multiple-comparison test. *p < 0.05, **p < 0.01, ***p < 0.001. ns, not significant.

To determine the appropriate concentration for *in vitro* experiments, the cytotoxicity of SPD toward IPEC-J2 cells was first evaluated using a CCK-8 assay. As shown in Figure 5A, SPD exhibited no significant cytotoxicity at concentrations ranging from 0 to 100 μM, whereas cell viability declined at concentrations of 200 μM and above. Accordingly, 25, 50, and 100 μM SPD were selected for subsequent experiments. Next, the protective effects of SPD against ETEC-induced cellular injury were investigated. ETEC challenge markedly reduced IPEC-J2 cell viability, while pretreatment with 50 and 100 μM SPD significantly attenuated this reduction; the 25 μM concentration showed no significant protective effect (Figure 5B). In parallel, ETEC infection induced robust inflammatory responses, as evidenced by significantly elevated secretion of IL-8 and TNF-*α*. SPD treatment dose-dependently suppressed the production of both cytokines, with the greatest inhibitory effect observed at 100 μM (Figures 5C,D). To further explore the underlying mechanism, the expression of proteins associated with AhR signaling and epithelial barrier integrity was examined by Western blotting (Figure 5E). Compared with the Control group, ETEC infection significantly decreased the protein levels of AhR, CYP1A1, ZO-1, and occludin. Notably, SPD treatment restored the expression of all four proteins in a concentration-dependent manner (Figures 5F–I). Collectively, these findings suggest that SPD alleviates ETEC-induced inflammatory injury in IPEC-J2 cells and preserves epithelial barrier integrity, which is associated with restoration of the AhR/CYP1A1 signaling pathway.

### Pharmacological inhibition of AhR abolishes the protective effects of spermidine against ETEC-induced intestinal injury

**Figure 6:**
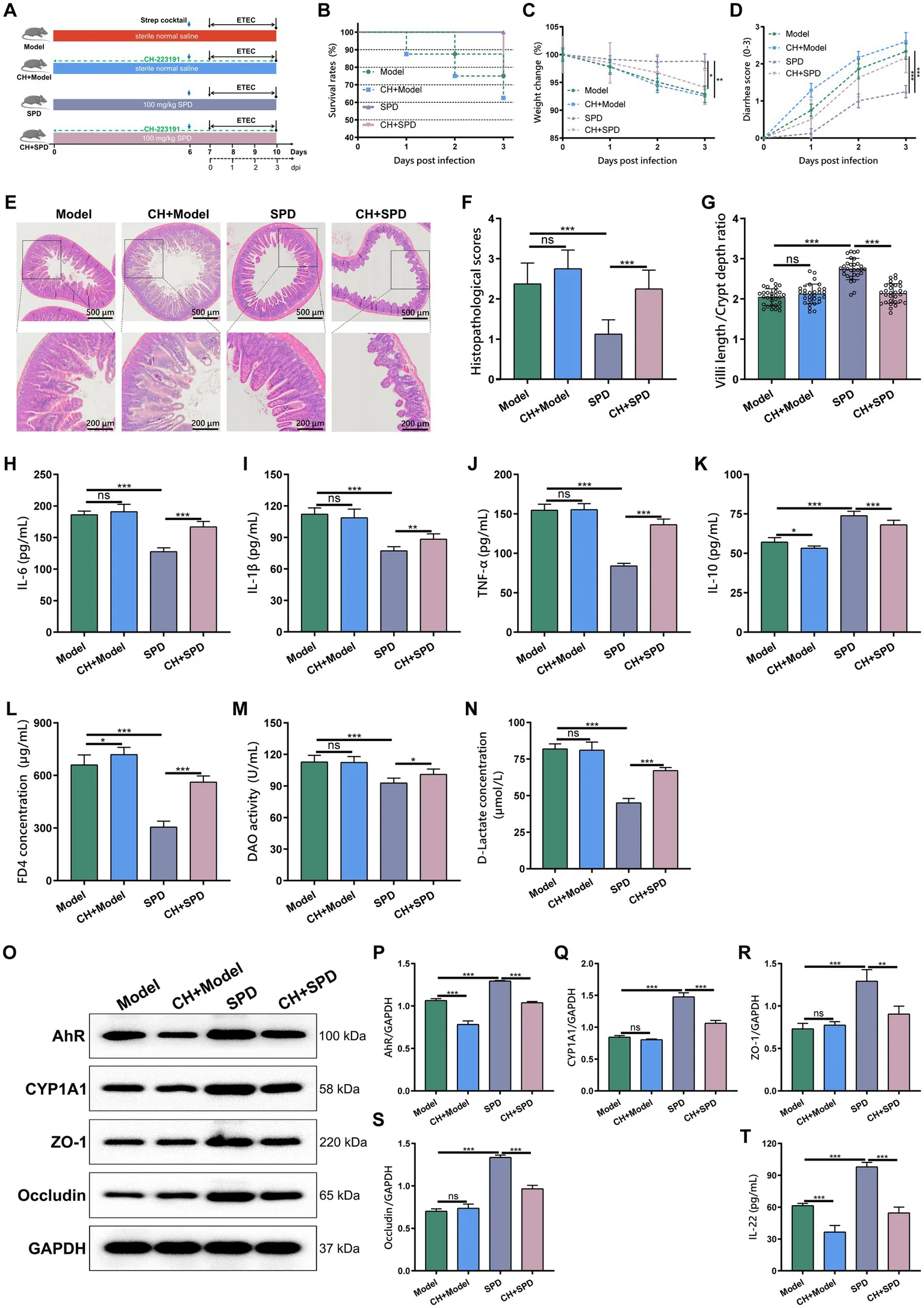
Pharmacological inhibition of AhR abolishes the protective effects of spermidine against ETEC-induced intestinal injury *in vivo***. (A)** Schematic illustration of the experimental design for AhR inhibition using CH-223191 in the ETEC infection model. **(B)** Survival curves of mice during the experimental period. **(C)** Changes in body weight following ETEC challenge. **(D)** Diarrhea scores. **(E)** Representative hematoxylin and eosin (H&E)-stained sections of the jejunum. Upper panels show low-magnification views (scale bar = 500 μm), and lower panels show corresponding higher-magnification images of the boxed regions (scale bar = 200 μm). **(F)** Histopathological injury scores. **(G)** Quantification of the villus-to-crypt (V/C) ratio. **(H–K)** Serum concentrations of IL-6 **(H)**, IL-1β **(I)**, TNF-α **(J)**, and IL-10 **(K)** determined by ELISA. **(L–N)** Assessment of intestinal permeability by serum FITC-dextran 4 kDa (FD4) **(L)**, diamine oxidase (DAO) activity **(M)**, and D-lactate **(N)**. **(O)** Representative Western blot images showing the expression of AhR, CYP1A1, ZO-1, and occludin in jejunal tissues. **(P–S)** Densitometric quantification of AhR **(P)**, CYP1A1 **(Q)**, ZO-1 **(R)**, and occludin **(S)** protein expression normalized to GAPDH. **(T)** Serum IL-22 levels measured by ELISA. Data are presented as mean ± SD. Statistical significance was determined by one-way ANOVA followed by Tukey’s multiple comparisons test. *p < 0.05, **p < 0.01, ***p < 0.001. ns, not significant.

To determine whether activation of the AhR pathway mediates the protective effects of spermidine *in vivo*, an additional experiment was performed using the selective AhR antagonist CH-223191 (Figure 6A). Compared with the SPD group, co-administration of CH-223191 largely abolished the beneficial effects of SPD on ETEC-induced disease progression. Although only modest differences in survival were observed among groups (Figure 6B), CH-223191 treatment markedly aggravated body weight loss and diarrhea severity and diminished the improvements conferred by SPD (Figures 6C,D). Histopathological analysis further supported these findings. Relative to the Model group, SPD administration substantially alleviated jejunal mucosal injury, as evidenced by reduced epithelial disruption and inflammatory infiltration, lower histological scores, and restoration of the villus-to-crypt ratio. In contrast, these protective effects were markedly attenuated following CH-223191 treatment, resulting in aggravated tissue damage and deterioration of intestinal architecture (Figures 6E–G). Consistent with the histological observations, SPD significantly suppressed systemic inflammation by reducing serum IL-6, IL-1β, and TNF-α levels while increasing IL-10 concentrations. Pharmacological inhibition of AhR largely reversed these anti-inflammatory effects, leading to elevated pro-inflammatory cytokine production and reduced IL-10 levels in the CH + SPD group (Figures 6H–K). Assessment of intestinal barrier function revealed that SPD markedly decreased serum FD4, DAO activity, and D-lactate levels compared with the Model group, indicating improved epithelial barrier integrity. However, these protective effects were substantially weakened by CH-223191 administration (Figures 6L–N). Likewise, Western blot analysis demonstrated that SPD restored the expression of AhR, its canonical downstream target CYP1A1, and the tight junction proteins ZO-1 and occludin, whereas concomitant treatment with CH-223191 significantly diminished their expression (Figures 6O–S). In addition, serum IL-22 levels were significantly increased following SPD treatment but were markedly reduced upon AhR inhibition (Figure 6T). Collectively, these findings demonstrate that pharmacological blockade of AhR largely abrogates the beneficial effects of spermidine on inflammation and intestinal barrier function, supporting the conclusion that activation of the AhR/CYP1A1/IL-22 signaling axis is a key mechanism underlying SPD-mediated protection against ETEC-induced intestinal injury.

### Spermidine reshapes the gut microbiota composition in ETEC-infected mice

**Figure 7:**
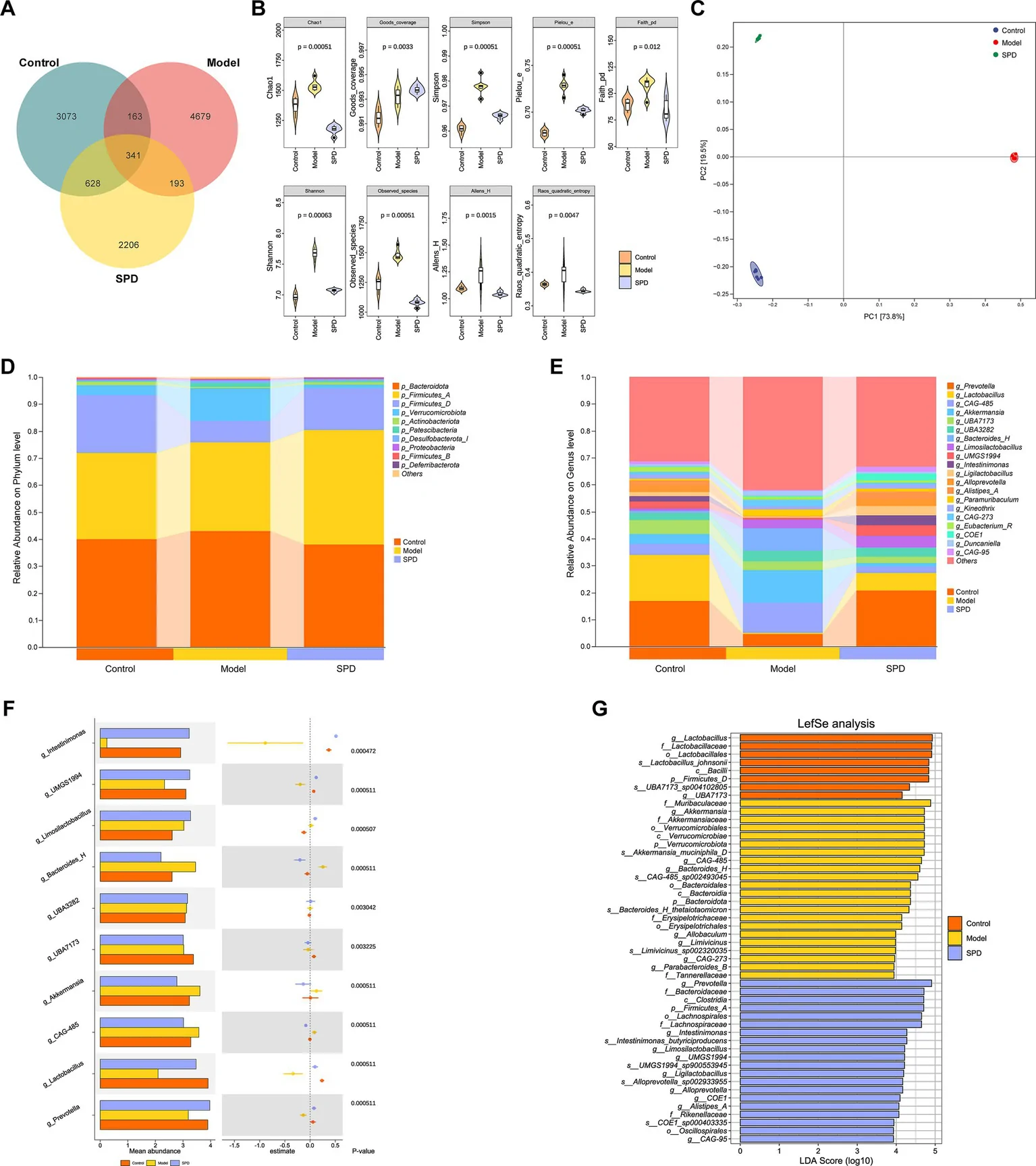
Spermidine remodels the gut microbiota composition in ETEC-infected mice. **(A)** Venn diagram showing the distribution of shared and unique amplicon sequence variants (ASVs) among the control, model, and SPD groups. **(B)** Alpha-diversity analysis of the gut microbiota, including representative richness and diversity indices (e.g., Chao1, Shannon, and Simpson). **(C)** Principal coordinate analysis (PCoA) based on microbial community composition. **(D)** Relative abundance of the dominant bacterial taxa at the phylum level. **(E)** Relative abundance of the major bacterial genera among experimental groups. **(F)** Differentially abundant genera identified by Kruskal–Wallis analysis. **(G)** Linear discriminant analysis effect size (LEfSe) identifying discriminative microbial taxa associated with each group. Data are presented as mean ± SD. Statistical significance was determined using the appropriate tests as described in the Methods section.

**Figure 8:**
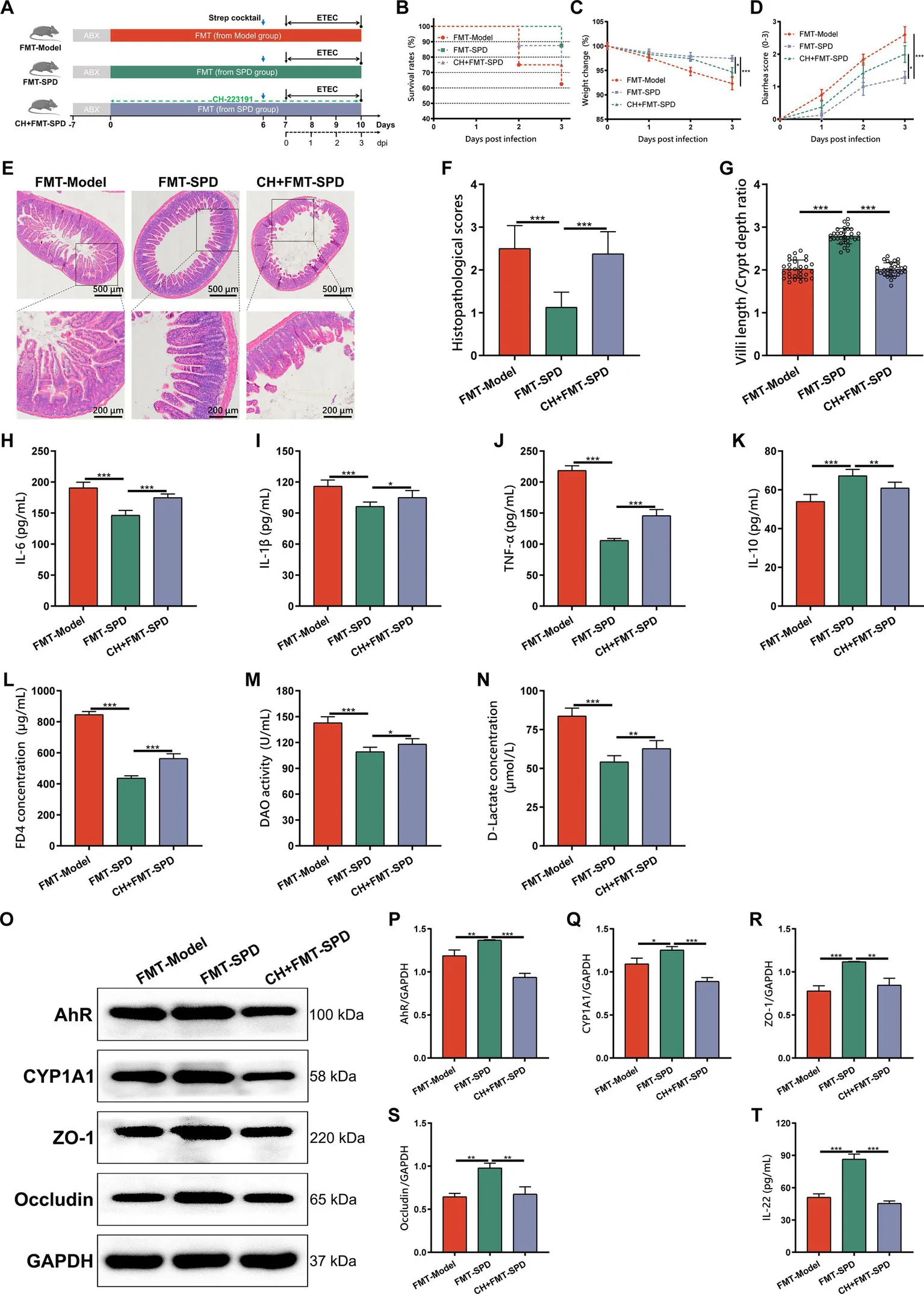
FMT from spermidine-treated donors protects against ETEC-induced intestinal injury through an AhR-dependent mechanism. **(A)** Experimental design of fecal microbiota transplantation (FMT) and CH-223191 intervention. **(B)** Kaplan–Meier survival curves. **(C)** Body weight changes during ETEC infection. **(D)** Diarrhea scores. **(E)** Representative hematoxylin and eosin (H&E)-stained jejunal sections. Upper panels show low-magnification views (scale bar = 500 μm), and lower panels show corresponding higher-magnification images of the boxed regions (scale bar = 200 μm). **(F)** Histopathological injury scores. **(G)** Villus-to-crypt (V/C) ratio. **(H–K)** Serum concentrations of IL-6 **(H)**, IL-1β **(I)**, TNF-α **(J)**, and IL-10 **(K)**. **(L–N)** Serum levels of FITC-dextran (FD4) **(L)**, diamine oxidase (DAO) activity **(M)**, and D-lactate **(N)**. **(O)** Representative Western blot images of AhR, CYP1A1, ZO-1, and occludin. **(P–S)** Quantification of AhR **(P)**, CYP1A1 **(Q)**, ZO-1 **(R)**, and occludin **(S)** protein expression. **(T)** Serum IL-22 concentration. Data are presented as mean ± SD. Statistical significance was determined by one-way ANOVA followed by Tukey’s multiple-comparison test. *p < 0.05, **p < 0.01, ***p < 0.001.

To investigate whether the protective effects of spermidine are associated with alterations in the gut microbiota, fecal samples from the Control, Model, and SPD groups were subjected to 16S rRNA gene sequencing. ASV analysis revealed substantial differences in microbial composition among the three groups (Figure 7A). The Control and Model groups shared 504 ASVs, whereas 969 ASVs were shared between the Control and SPD groups, indicating that the microbial community in SPD-treated mice was more similar to that of healthy controls than to ETEC-infected mice. Alpha-diversity analysis demonstrated that ETEC infection significantly altered microbial richness and community diversity. Compared with the Control group, the Model group exhibited higher *α*-diversity indices (e.g., Chao1, Shannon, and Simpson), whereas SPD treatment partially normalized these alterations, yielding a microbial profile more similar to that of healthy controls (Figure 7B). Principal coordinate analysis (PCoA) further demonstrated distinct clustering of the three groups, indicating significant differences in overall microbial community structure (Figure 7C). Although the SPD group remained clearly separated from the Control group, the distance between these two groups was substantially smaller than that between the Control and Model groups, suggesting that spermidine partially reversed the microbial dysbiosis induced by ETEC infection. At the phylum level, *Firmicutes* and *Bacteroidetes* remained the predominant bacterial phyla across all groups (Figure 7D). Compared with the Control group, ETEC infection was associated with increased relative abundances of *Bacteroidetes* and *Verrucomicrobiota*, accompanied by decreased abundances of *Firmicutes_D* and *Actinobacteriota*. SPD intervention partially restored these ETEC-induced alterations, shifting the microbial composition toward that observed in healthy controls. At the genus level, ETEC challenge reduced the relative abundances of *Prevotella* and *Lactobacillus*, while increasing the abundances of *CAG-485* and *Akkermansia*. These changes were markedly reversed following SPD treatment (Figure 7E). Consistently, Kruskal–Wallis analysis of the top differential genera showed that SPD selectively enriched *Prevotella*, *Lactobacillus*, *UMGS1994*, and *Intestinimonas*, while reducing the abundances of *CAG-485*, *Akkermansia*, and *Bacteroides_H* compared with the Model group (Figure 8F). Linear discriminant analysis effect size (LEfSe) further identified distinct microbial signatures among experimental groups (Figure 8G). Lactobacillus, together with its higher taxonomic ranks *Lactobacillaceae* and *Lactobacillales*, was significantly enriched in the Control group, whereas *Muribaculaceae* was a characteristic taxon in the Model group. In contrast, the SPD group was characterized by significant enrichment of *Prevotella*, *Bacteroidaceae*, *Clostridia*, and *Firmicutes_A*, indicating that spermidine promotes a distinct microbial configuration associated with protection against ETEC-induced intestinal injury. Collectively, these findings demonstrate that spermidine markedly remodels the gut microbiota in ETEC-infected mice, partially restoring infection-associated microbial dysbiosis and selectively enriching bacterial taxa that may contribute to its protective effects on intestinal homeostasis.

### Fecal microbiota transplantation from spermidine-treated donors confers protection against ETEC-induced intestinal injury through an AhR-dependent mechanism

To investigate whether the protective effects of spermidine are transferable through modulation of the gut microbiota, FMT was performed using donor mice from the Model or SPD groups, followed by ETEC challenge (Figure 8A). Recipient mice receiving microbiota from SPD-treated donors (FMT-SPD) exhibited markedly improved clinical outcomes compared with those receiving microbiota from Model donors (FMT-Model), including reduced body weight loss, lower diarrhea scores, and improved survival during the infection period (Figures 8B–D). Histopathological analysis further demonstrated that FMT-SPD effectively alleviated ETEC-induced intestinal injury, as evidenced by preservation of villus architecture, reduced inflammatory cell infiltration, lower histological scores, and restoration of the villus-to-crypt ratio (Figures 8E–G). In parallel, FMT-SPD significantly attenuated systemic inflammation by decreasing serum IL-6, IL-1β, and TNF-α levels while increasing IL-10 concentrations (Figures 8H–K). Moreover, mice receiving microbiota from SPD-treated donors displayed improved intestinal barrier function, reflected by reduced serum FD4, DAO activity, and D-lactate levels compared with the FMT-Model group (Figures 8L–N). Mechanistically, FMT from SPD-treated donors markedly increased the protein expression of AhR, CYP1A1, ZO-1, and occludin and significantly elevated circulating IL-22 levels (Figures 8O–T), indicating restoration of AhR signaling and epithelial barrier integrity. Importantly, pharmacological inhibition of AhR with CH-223191 largely abolished these beneficial effects. Compared with the FMT-SPD group, mice in the CH + FMT-SPD group exhibited aggravated clinical symptoms and intestinal pathology, enhanced inflammatory responses, impaired barrier function, reduced expression of AhR/CYP1A1 and tight junction proteins, and decreased serum IL-22 levels. Collectively, these findings demonstrate that transplantation of microbiota from SPD-treated donors is sufficient to confer protection against ETEC-induced intestinal injury in recipient mice. The loss of this protection following AhR inhibition further supports that the beneficial effects of SPD-associated microbiota are mediated, at least in part, through activation of the AhR/CYP1A1/IL-22 signaling pathway.

## Discussion

ETEC remains one of the leading causes of infectious diarrhea and intestinal inflammation in both humans and livestock, primarily through disruption of epithelial barrier integrity and induction of excessive host inflammatory responses (von Mentzer and Svennerholm, 2024; Zhang et al., 2022). Although SPD has been implicated in cellular homeostasis, autophagy, and immune regulation, its role in ETEC-induced intestinal injury and the underlying mechanisms have remained largely unexplored (Eisenberg et al., 2009; Madeo et al., 2018). In the present study, we demonstrate that SPD pretreatment markedly protects against ETEC-induced intestinal damage by improving clinical outcomes, suppressing inflammatory responses, preserving epithelial barrier integrity, and remodeling the gut microbiota. More importantly, pharmacological inhibition of AhR markedly attenuated these protective effects, while FMT from SPD-treated donors partially transferred the beneficial phenotype to recipient mice. Together, our findings support a model in which SPD protects against ETEC-induced intestinal injury, at least partly, through microbiota-associated activation of the AhR/CYP1A1/IL-22 signaling axis.

Maintenance of intestinal barrier integrity is essential for limiting pathogen invasion and preventing excessive mucosal inflammation (Shin and Kim, 2018). As the predominant clinical manifestation of ETEC infection, diarrhea results primarily from toxin-mediated disruption of epithelial ion transport, increased intestinal permeability, and impaired mucosal barrier function. Consistent with previous reports describing ETEC-induced epithelial disruption (Gao et al., 2025), our study showed that infected mice developed pronounced weight loss, diarrhea, histopathological injury, and reduced villus-to-crypt ratios, accompanied by elevated circulating inflammatory cytokines and increased intestinal permeability. SPD treatment significantly attenuated these pathological changes, restored the expression of the tight junction proteins ZO-1 and occludin, and reduced serum FD4, DAO, and D-lactate levels, indicating preservation of epithelial barrier function. These *in vivo* findings were further corroborated in ETEC-challenged IPEC-J2 cells, where SPD improved cell viability, reduced IL-8 and TNF-α production, and restored tight junction protein expression, supporting a direct protective effect on intestinal epithelial cells. The pathogenicity of ETEC is largely attributed to its major virulence factors, including LT and ST, which disrupt intestinal ion transport and fluid secretion through distinct intracellular signaling pathways (Xia et al., 2015). Although the present study did not directly measure ETEC toxin production or toxin-specific signaling events, the beneficial effects of SPD on epithelial barrier integrity and inflammatory regulation suggest that SPD may alleviate toxin-associated intestinal dysfunction by strengthening host defense mechanisms rather than directly neutralizing bacterial toxins. Future studies are warranted to determine whether SPD influences LT/ST expression, receptor engagement, or downstream toxin-mediated signaling cascades.

Excessive activation of the TLR4/MyD88/NF-κB pathway represents a central mechanism driving ETEC-induced inflammation (Li et al., 2024). Consistent with previous studies, ETEC infection markedly increased the expression of TLR4 and MyD88, accompanied by enhanced phosphorylation of NF-κB p65 and elevated production of inflammatory cytokines. SPD effectively suppressed activation of this signaling cascade and restored serum IL-10 levels toward the physiological range observed in healthy controls, suggesting that SPD contributes to the re-establishment of immune balance during bacterial infection. Considering that TLR4 activation is primarily initiated by bacterial LPS, the inhibitory effect of SPD on this pathway may not necessarily result from direct interaction with TLR4. Instead, SPD may indirectly regulate TLR4 signaling by reducing microbial stimulation, maintaining epithelial barrier integrity, and reshaping the gut microbiota. Moreover, modulation of downstream adaptor molecules, including MyD88, may also contribute to the attenuation of inflammatory signaling. Therefore, the precise molecular relationship between SPD and TLR4 pathway components requires further investigation.

One of the most significant findings of this study is the identification of AhR signaling as a critical mediator of SPD activity. AhR functions as an important environmental sensor regulating mucosal immunity, epithelial renewal, and host–microbiota interactions (Monteleone et al., 2011). Activation of AhR induces canonical target genes such as CYP1A1 and promotes IL-22 production, thereby enhancing epithelial repair and barrier maintenance (Gronke et al., 2019; Miyamoto et al., 2024). Our data demonstrated that SPD restored AhR and CYP1A1 expression and significantly elevated circulating IL-22 levels in ETEC-infected mice. Importantly, pharmacological blockade of AhR with CH-223191 substantially diminished the beneficial effects of SPD on clinical symptoms, inflammatory responses, epithelial barrier function, and tight junction protein expression. These observations indicate that AhR/CYP1A1/IL-22 signaling contributes substantially to the protective effects of SPD during ETEC infection.

Emerging evidence indicates that gut microbial metabolites are major endogenous regulators of AhR activity (Rothhammer et al., 2016), suggesting that microbiota remodeling may represent an upstream mechanism linking SPD to AhR activation. Consistent with this concept, 16S rRNA sequencing revealed that SPD substantially reshaped the gut microbial community following ETEC infection. Principal coordinate analysis demonstrated that SPD partially restored the infection-induced microbial dysbiosis toward a configuration resembling healthy controls. Moreover, SPD selectively increased the relative abundance of several genera, including *Prevotella*, *Lactobacillus*, *UMGS1994*, and *Intestinimonas*, while reducing taxa such as *CAG-485*, *Akkermansia*, and *Bacteroides_H*. Although the precise functional contributions of individual taxa require further investigation, these compositional changes suggest that SPD establishes a microbial ecosystem more favorable for intestinal homeostasis and mucosal protection.

To determine whether microbiota remodeling contributes causally to SPD-mediated protection, we further performed FMT experiments. Notably, recipient mice receiving microbiota from SPD-treated donors exhibited significant improvements in disease severity, inflammatory cytokine profiles, epithelial barrier function, and AhR/CYP1A1 signaling despite never receiving SPD directly. This transferable protective phenotype provides compelling evidence that SPD exerts part of its beneficial effects through modulation of the gut microbiota. Furthermore, administration of CH-223191 largely abolished the protection conferred by SPD-derived microbiota, indicating that activation of AhR remains indispensable even after microbiota transfer. These findings suggest that gut microbiota remodeling may act upstream of AhR signaling activation and provide mechanistic support for the involvement of microbiota–host interactions in SPD-mediated intestinal protection.

**Figure 9:**
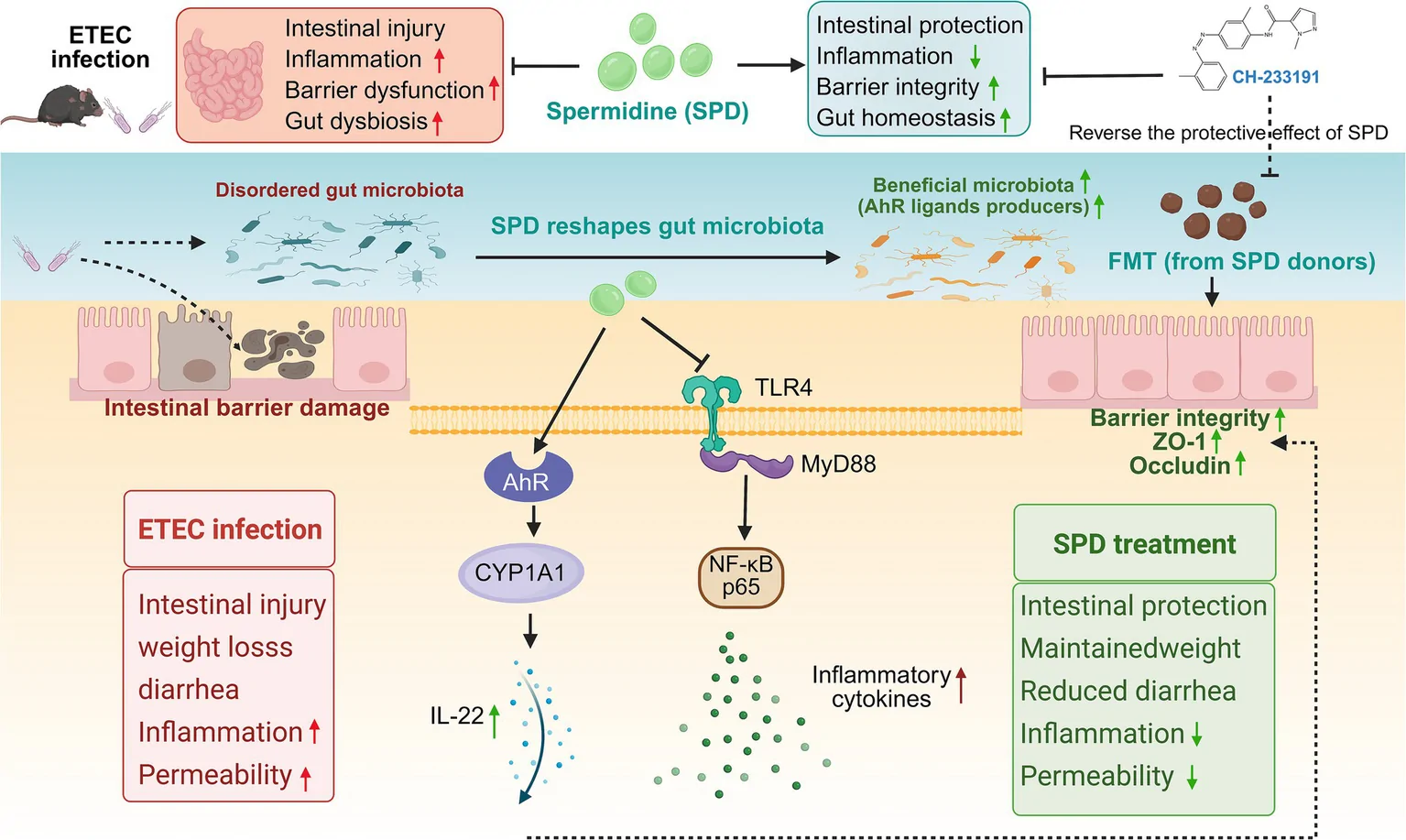
Proposed mechanism underlying the protective effects of spermidine against ETEC-induced intestinal injury. Spermidine remodels the gut microbiota and activates the AhR/CYP1A1/IL-22 signaling pathway, thereby suppressing inflammation, preserving intestinal barrier integrity, and protecting against ETEC-induced intestinal injury.

Taken together, our study supports a model in which SPD remodels the gut microbial ecosystem, thereby promoting activation of the AhR/CYP1A1/IL-22 pathway, reinforcing epithelial barrier integrity, suppressing excessive inflammatory responses, and ultimately protecting against ETEC-induced intestinal injury (Figure 9). Given that ETEC represents a major cause of neonatal diarrhea and post-weaning diarrhea in livestock, particularly piglets, SPD supplementation may represent a promising nutritional strategy for improving intestinal resilience and reducing the impact of ETEC-associated diseases in animal production. Further studies in livestock models are required to evaluate its practical applicability and efficacy under farm-relevant conditions.

Although our findings provide important insights into the protective effects of SPD against ETEC-induced intestinal injury, several limitations should be acknowledged. First, although human ETEC infection is predominantly associated with acute secretory diarrhea rather than severe intestinal tissue damage, experimental ETEC challenge models are widely used to investigate pathogen-induced epithelial barrier dysfunction, inflammatory responses, and host defense mechanisms. Therefore, our findings should be interpreted primarily in the context of intestinal barrier protection and immune regulation during enteric bacterial infection, with potential implications for both human and veterinary applications. Second, the present study did not include an SPD-only group under physiological conditions. Therefore, although our data demonstrate that SPD effectively counteracts ETEC-induced pathological changes, we cannot completely exclude the possibility that SPD may independently influence basal inflammatory status, intestinal morphology, or gut microbiota composition in the absence of infection. Future studies incorporating healthy animals treated with SPD alone will further clarify the intrinsic immunomodulatory effects of SPD and its contribution to intestinal homeostasis. Third, although our findings support the involvement of gut microbiota remodeling in SPD-mediated AhR pathway activation, the precise molecular mechanisms linking microbial alterations to host signaling remain incompletely defined. Specifically, the microbial metabolites responsible for AhR activation, particularly tryptophan-derived AhR ligands, were not directly quantified. In addition, although FMT and AhR inhibition experiments demonstrate the contribution of microbiota–AhR interactions to SPD-mediated protection, they do not identify the specific bacterial taxa or metabolites responsible for this regulation. Future studies integrating metabolomics, metagenomics, gnotobiotic models, targeted microbial colonization/depletion approaches, and direct IL-22 intervention will further elucidate the molecular mechanisms underlying SPD-mediated intestinal protection.

## Conclusion

In conclusion, this study demonstrates that spermidine protects against ETEC-induced intestinal injury by attenuating excessive inflammation and preserving epithelial barrier integrity. Our findings suggest that spermidine-mediated gut microbiota remodeling is associated with activation of the AhR/CYP1A1/IL-22 signaling pathway, which may contribute to enhanced tight junction expression and restoration of intestinal homeostasis. The protective phenotype was partially transferable through FMT and was attenuated by pharmacological inhibition of AhR, supporting the involvement of the microbiota–AhR axis in spermidine-mediated intestinal protection. These findings provide new insights into the immunomodulatory effects of spermidine and highlight its potential as a nutritional strategy for improving host resistance against enteric bacterial infections.

## Data Availability

The original contributions presented in the study are included in the article/supplementary material, further inquiries can be directed to the corresponding author.
